# Making Collections

**Published:** 1914-06

**Authors:** 


Making Collections.—In many ways physicians are very poor business men. Of course, there are exceptions, but comparatively few of them ever accumulate very much money, and many of them leave uncollected more of their fees than they ever collect. It is all right to respond to cases of charity, and to do all that can reasonably be done for those who are in real need, but there is no rule of professional ethics that requires or that even justifies a physician to neglect to secure reasonable compensation for services rendered. The physician should adopt business methods just as they are in use in business establishments of the country, and they should use every reasonable effort to collect what is due them.

It is a mistaken idea that they lose friends by such methods. It is true that the physician occupies a very intimate and close relationship with those whom he serves, but this should not cause him to hesitate to promptly present bills for his services, and when they are not paid to present them again and again, as business men do. You can rest assured that if you owe your grocer, your dry goods man, or your plumber, however close his relation may be to you, your bill will be presented and payment will be urged. Some people impose upon physicians because they know their professional relations to them often cause them to hesitate to press collections.

If you have been negligent in these matters, make a new start in the right direction. Collect your bills promptly. Pay your debts promptly. Hold your head up and be respected by the people of your community.

